# *Toxoplasma gondii* in dromedary camels (*Camelus dromedarius*) in Egypt: a comparative seroepidemiological study in Upper and Lower Egypt

**DOI:** 10.3389/fvets.2024.1508496

**Published:** 2025-01-20

**Authors:** Ehab Kotb Elmahallawy, Nady Khairy Elbarbary, David Cano-Terriza, Tomás Fajardo, Nada Oudah Albalawi, Débora Jiménez-Martín, Marwa M. I. Ghallab, Ahmed Gareh, Refaat Ras, Isabelle Villena, Sabry A. S. Sadek, Hajar AlQadeeb, Hind Alzaylaee, Sonia Almería, Ignacio García-Bocanegra

**Affiliations:** ^1^Departamento de Sanidad Animal, Grupo de Investigación en Sanidad Animal y Zoonosis (GISAZ), Universidad de Córdoba, Córdoba, Spain; ^2^Department of Zoonoses, Faculty of Veterinary Medicine, Sohag University, Sohag, Egypt; ^3^Department of Food Hygiene, Faculty of Veterinary Medicine, Aswan University, Aswan, Egypt; ^4^CIBERINFEC, ISCIII CIBER de Enfermedades Infecciosas, Instituto de Salud Carlos III, Madrid, Spain; ^5^Department of Biology, Faculty of Science, Taibah University, Alula, Saudi Arabia; ^6^Department of Medical Parasitology, Faculty of Medicine, Kafrelsheikh University, Kafr El Sheikh, Egypt; ^7^Department of Parasitology, Faculty of Veterinary Medicine, Aswan University, Aswan, Egypt; ^8^Department of Parasitology, Faculty of Veterinary Medicine, Zagazig University, Zagazig, Egypt; ^9^Department of Microbiology and Parasitology, Faculty of Veterinary Medicine, Badr University in Cairo (BUC), Badr City, Egypt; ^10^University of Reims Champagne-Ardenne, UR 7510, National Reference Centre for Toxoplasmosis, Laboratory of Parasitology, Reims Hospital, Reims, France; ^11^Department of Zoonotic Diseases, National Research Centre, Giza, Egypt; ^12^Department of Medical Laboratory, College of Applied Medical Sciences, Prince Sattam Bin Abdulaziz University, AlKharj, Saudi Arabia; ^13^Department of Biology, College of Science, Princess Nourah bint Abdulrahman University, Riyadh, Saudi Arabia; ^14^Virology and Parasitology Branch, Division of Food and Environmental Safety, Office of Applied Microbiology and Technology (OAMT), Office of Laboratory Operations and Applied Sciences (OLOAS), Food and Drug Administration, Department of Health and Human Services, Laurel, MD, United States

**Keywords:** *Toxoplasma gondii*, camels, serosurvey, modified agglutination test, Egypt

## Abstract

Toxoplasmosis remains a prevalent parasitic zoonosis worldwide, raising public health concerns. The global information available regarding the role of camels in the epidemiology of *Toxoplasma gondii* is still limited. This study aimed to assess the seroprevalence of *T. gondii* in dromedary camels (*Camelus dromedarius*) from northern and southern Egypt. A total of 513 serum samples were obtained from camels across Cairo (Lower Egypt) and Aswan (Upper Egypt) governorates. The Modified Agglutination Test (MAT) was performed to screen for anti-*T. gondii* antibodies. The overall seroprevalence was 13.84% (71/513; 95CI%:10.85–16.83). The bivariate analysis showed that animals aged 4–8 years (13.84%, 36/260) and older than 8 years (18.45%, 31/168) showed significantly higher seropositivity compared to those young individuals (≤ 4 years old) (*p* = 0.011). Additionally, the multiple logistic regression analysis highlighted the geographic region as a potential risk factor for *T. gondii* exposure. Thus, camels from Lower Egypt had significantly higher seroprevalence of *T. gondii* (19.92%, 51/256) compared to those from Upper Egypt (7.78%; 20/257; *p* < 0.001; odds ratio [OR] = 2.94; 95% CI: 1.70–5.10). Our results provide evidence of moderate, widespread, and heterogeneous spatial distribution of *T. gondii* among camel populations in Egypt, which might have important implications for animal and public health in that country. Surveillance and control programs should be implemented to reduce the risk of exposure of *T. gondii* in camels.

## 1 Introduction

Toxoplasmosis is recognized as one of the most significant and globally widespread parasitic zoonoses (1). The disease is caused by the apicomplexan intracellular protozoan, *Toxoplasma gondii*, which infects all warm-blooded species (2). The distribution of this parasite varies widely across different regions worldwide, reliant on ecological, climatic and environmental factors (3). *Toxoplasma gondii* is an opportunistic parasite which relies on both definitive and intermediate hosts to complete its cycle. The sexual stage occurs in the intestine of the definitive hosts, which are members of the *Felidae* family (4). This protozoan undergoes an asexual stage in various tissues of a wide range of warm-blooded animals, including humans, which serve as intermediate hosts (4). Both definitive and intermediate hosts may get infected via one of the three main stages: sporulated oocysts, tachyzoites, or bradyzoites over three major routes: (a) horizontally through oral ingestion of sporulated oocysts from contaminated food and water, (b) horizontally over ingestion of bradyzoite tissue cysts in undercooked meat and offal of intermediate host, and (c) vertically through transplacental transmission of tachyzoites and milk of infected hosts (5).

Dromedary camels (*Camelus dromedarius*) are versatile animals, serving multiple purposes for humans, such as transportation and providing milk, meat, and hair. They hold significant value in nomadic or pastoralist communities residing in arid or semi-arid regions. The production and management systems of camels in Egypt are predominantly small-scale, with about 120,000 camels censed (6). Egypt also relies heavily on imports from neighboring African countries, particularly Sudan, to meet its demand for camels. However, camel production in Egypt is challenged by unsuitable management systems and a wide range of transmissible diseases affecting this species (7–9). Among them, toxoplasmosis has shown to have a notable impact on this species (10–12). *Toxoplasma gondii* infection in camels encompasses a wide range of clinical signs, including general signs like fever, lethargy and weight loss, neurological and respiratory symptoms and reproductive disorders such as abortion and stillbirth (10). Also, food products from camels are frequently being consumed by humans, and therefore might represent a major source of zoonotic infection (13).

Previous studies conducted in Egypt have shown varying *T. gondii* exposure in camel populations across the country with seroprevalence values ranging between 0.81 and 96.42% [Table 1; (3, 8, 14–31)]. However, survey studies comparing seroepidemiological data in Upper and Lower Egypt are still very limited. Therefore, the aims of the present study were to provide an update about the serological occurrence of *T. gondii* in dromedary camels in Egypt and to determine the seroprevalence in different populations from Lower and Upper Egypt.

**Table 1:** Seroprevalence of *Toxoplasma gondii* reported in dromedary camels (*Camelus dromedaries*) in Egypt.

Location	Governorate	Detection method	Cut-off value	Prevalence % (no. pos./total)	References
Lower Egypt	Different	IFA	NS	6.12 (3/49)	Maronpot and Botros (14)
	Ismailia	DT	1:8	67.44 (29/43)	Rifaat et al. (15)
	Menoufiya	DT	1:16	18.75 (15/80)	Michael et al. (16)
	Marsa Matrouh	DT	1:16	50.00 (40/80)	Michael et al. (16)
	Menoufiya	DT	1:8	63.01 (46/73)	Rifaat et al. (17)
	Sharkia	IHA	NS	26.31 (5/19)	El Ridi et al. (18)
	Gharbia	IHA	1:40	48.83 (126/258)	Ibrahim et al. (19)
	Cairo	MAT	1:25	17.46 (29/166)	Hilali et al. (20)
	Cairo	MAT	1:25	18.00 (27/150)^a^	Shaapan et al. (21)
				20.00 (30/150)^b^ 27.33 (41/150)^c^ 30.66 (46/150)^d^	
	Cairo	ELISA	NS	66.66 (40/60)	Toaleb et al. (22)
	Matrouh	LAT	NS	60.37 (32/53)	Osman et al. (23)
	Cairo and Giza	ELISA	NS	26.47 (9/34)	Elfadaly et al. (24)
	Qalyubia	LAT ELISA	NS NS	5.00 (6/120) 52.50 (63/120)	Ahmed et al. (8)
	Cairo and Giza	IHA	1:8	57.50 (115/200)	Gerges et al. (25)
	Marsa Matrouh	ELISA	NS	64.51 (80/124)	Khattab et al. (26)
	Kafr ElSheikh	ELISA	NS	39.16 (47/120)	Selim et al. (3)
	Qalyubia	ELISA		45.00 (54/120)	
	Marsa Matrouh	ELISA		54.66 (82/150)	
Upper Egypt	Assiut	DT	1:16	15.00 (12/80)	Michael et al. (16)
	Assiut	DT	1:4	24.40 (30/119)	Fahmy et al. (27)
	Assiut	LAT ELISA	1:2 1:2	35.71 (20/56) 96.42 (54/56)	Kuraa et al. (28)
	Aswan	LAT	NS	32.43 (12/37)	Sameeh et al. (29)
	Abu Simbel (Aswan)	ELISA	NS	2.17 (2/92)	Fereig et al. (43)
	Shalateen (Red Sea)	ELISA		31.52 (116/368)	
Both regions	Beni Suef, Giza, Menoufia, Alexandria, Sharqia, Matruh, and Faiyum	ELISA	NS	36.58 (15/41)	Zeedan et al. (31)

ELISA, enzyme-linked immunosorbent assay; DAT, direct agglutination test; IHA, indirect hemagglutination; LAT, latex agglutination test; MAT, modified agglutination test; IFAT, indirect fluorescent antibody test; FMI, following manufacturer instructions; NS, not specified.

a Using RH strain.

b Using local equine strain.

c Using local camel strain.

d Using local sheep strain.

## 2 Materials and methods

### 2.1 Study area

**Figure 1:**
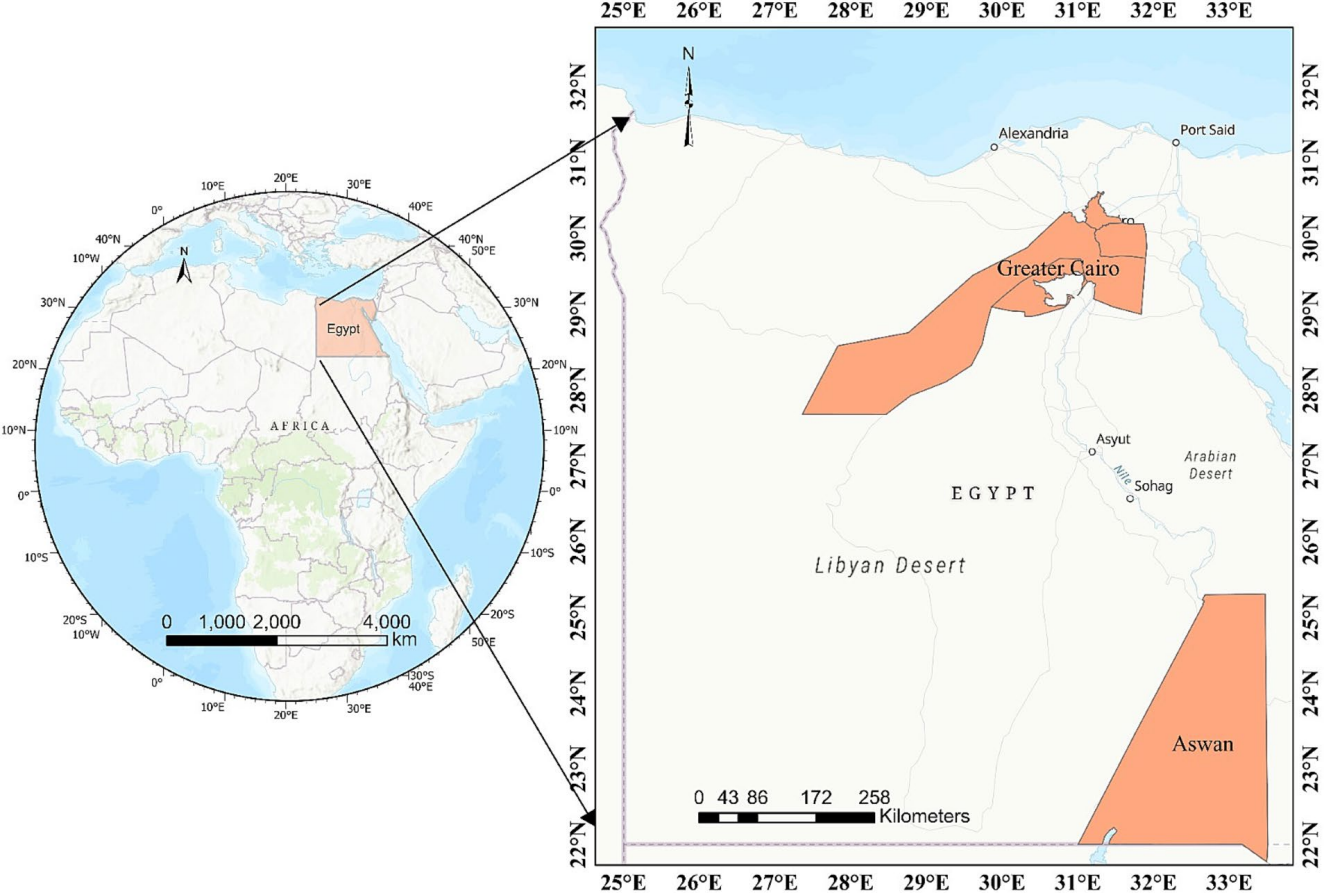
A map of Egypt delineating the geographical locations where camel samples were collected in Upper and Lower Egypt.

A cross-sectional study was conducted in the two major governorates of Egypt, namely Cairo and Aswan, representing Lower and Upper Egypt, respectively (Figure 1). Cairo governorate serves as the capital and largest city of the country. The total area is 3,048.676 km^2^, the inhabited space is 188.982 km^2^, located near the Nile Delta extends 25 km on the western bank of the Nile River in Egypt at 30° 02′ 30″ N, 031° 14′ 07″ E. Egypt’s capital has a mild-to-hot climate for most of the year, with maximum temperatures around 34°C in summer and 18°C in winter. Aswan governorate covers a total area of 62,726 km^2^, with an inhabited area of 12,203 km^2^. It is located in southern Egypt at coordinates 24° 5′ 20.1768″ N and 32° 53′ 59.3880″ E, just north of the Aswan Dam on the east bank of the Nile near the first cataract. The climate of Aswan is very hot and dry in summer and might exceed 41°C, while winter is relatively mild with an average of 26°C and dry and rainfall is non-existent except sometimes in the month of August. Rainfall in Egypt is very low across the country, with an average annual precipitation of less than 80 mm, occurring primarily during the winter months.

### 2.2 Animals and samples

Between January and May 2023, 513 blood samples were randomly collected from apparently healthy dromedary camels from different regions of Lower and Upper Egypt. Samples were collected from local markets and veterinary campaigns. A total of 513 samples were collected from two regions: 256 from Cairo and 257 from the Aswan governorate. The age of animals ranged from 3 to 15 years, with a median age of 7 years, and were primarily used for fieldwork and meat production. A total of 10 mL blood samples were collected from each animal by puncturing the jugular vein. Plain tubes without anticoagulant were used, along with sterile syringe needles, for the collection process. All samples were kept in portable coolers containing polyethylene ice packs then sent to the laboratories of Zoonotic diseases at National Research Centre (Egypt), for samples collected from Cairo governorate, and Department of Food Hygiene, Aswan University for samples from Aswan governorate. Sera were obtained by centrifugation at 1,107 rcf for 15 min then the supernatant was transferred to a new Eppendorf tube and kept at −20°C until analysis. Data regarding the age, sex, and region of each animal were collected, whenever possible. The ages of the examined camels were estimated and documented based on information provided by their owners and an assessment of the animals’ dentition (32, 33). Camels were classified into three age categories: young (≤4 years old), adults (between 4 and 8 years old), and elders (>8 years old), as described in previous studies (3, 13).

### 2.3 Serological assessment

Serum samples were serologically examined for the presence of anti-*T. gondii* antibodies, specifically IgG, using modified agglutination test (MAT), which employs formalin-fixed tachyzoites as described elsewhere (34, 35). Antigen of *T. gondii* was supplied by NRC on toxoplasmosis (Reims). Sera with titers ≥1:25, were considered positive, which serves as the established cut-off for *T. gondii* seropositivity, as previously considered for these animal species (36, 37). Sera that initially tested positive at a dilution of ≥1:25 were subsequently retested at dilutions of 1:25, 1:50, 1:100, and ≥1:500.

### 2.4 Statistical analysis

To assess the seroprevalence of *T. gondii*, we computed the proportion of seropositive samples relative to the total number of camels examined, accompanied by a 95% confidence interval (95%CI). Associations between explanatory variables [age (categorized as ≤4 years, 4–8 years, and >8 years), sex (male and female), and region (Upper and Lower Egypt)] and serological results were analyzed with the use of a Pearson’s chi-square test and by Fisher’s exact test when observations/category were < 6. Variables with a *p*-value below 0.10 were selected to be included in the multivariate analysis. Collinearity between pairs of variables was tested using the Cramer’s V, selecting the variable with the highest biological plausibility. Finally, a multiple logistic regression was carried out to study the effect of potential explanatory variables previously selected in the bivariate analysis (38). All analyses were conducted using SPSS software, version 25.0^®^ (Statistical Package for the Social Sciences, Inc., Chicago, IL, United States), and statistical significance was set at *p*-value < 0.05.

## 3 Results

Anti-*T. gondii* antibodies were detected in 71 (13.84%; 95CI%:10.85–16.83) of the 513 camels analyzed. Seropositive animals exhibited varying anti-*T. gondii* antibodies titers: 1:25 in 14 animals (2.73%), 1:50 in 11 (2.14%), 1:100 in 28 (5.46%), and ≥1:500 in 18 (3.51%). The distribution of *T. gondii* seropositivity according to region, age and sex is shown in Table 2. The prevalence of anti-*T. gondii* antibodies was significantly higher in camels from Lower Egypt (19.92%; 51/256; 95 CI%: 15.03–24.81) compared to those from Upper Egypt (7.78%; 20/257; 95CI%: 4.51–11.06) (*p* < 0.001). Significantly higher seropositivity was also found in older (18.45%; 31/168) and adult (13.84%, 36/260) animals compared to young individuals (4.70%, 4/85) (*p* = 0.010). The multivariate analysis identified geographic region as a potential risk factor for *T. gondii* exposure, with significantly higher seropositivity observed in camels from northern Egypt compared to those from southern Egypt (*p* < 0.001, OR = 2.94; 95% CI: 1.70–5.10).

**Table 2:** Univariable analysis of risk factors associated to *T. gondii* infection in camels.

Variable	Category	Positives/total*	Seroprevalence (%)	*p* value
Region	Lower Egypt	51/256	19.92	<0.001
	Upper Egypt	20/257	7.78	
Age	≤4 years	4/85	4.70	0.011
	4–8 years	36/260	13.84	
	>8 years	31/168	18.45	
Sex	Male	66/478	13.80	0.937
	Female	5/35	14.28	

## 4 Discussion

Toxoplasmosis presents significant clinical and economic challenges in both human and animal health. In humans, it typically manifests with flu-like symptoms, including fever, lymphadenopathy, and ocular issues. The infection can also lead to multiple neurological and reproductive disorders (39). While in livestock, it is a leading cause of abortion, stillbirth, and weak offspring, all of which critically impact productivity and economic sustainability (40). Given this significant medical and veterinary implications, regular and periodic updates of seroepidemiological data on *T. gondii* among different reservoirs are pivotal for establishing a baseline for its control. To the best of the authors knowledge, the current study represents the most extensive serological investigation conducted among dromedary camel populations in Egypt. The overall seroprevalence obtained (13.84%) falls within the values previously reported in this species in Egypt (Table 1). Nevertheless, comparisons among studies should be made with caution given the differences in number of animals examined, serological methods employed and management and environmental factors.

In the present work, we determine the seroprevalence of *T. gondii* in the northern and southern part of Egypt, evidencing a heterogeneous spatial distribution and exposure to this parasite across the country. The risk factor analysis revealed that camels from Lower Egypt had a 2.9-fold higher risk of *T. gondii* exposure compared to those in Upper Egypt, suggesting greater circulation of this parasite in the northern region of the country. As depicted, significantly higher seroprevalence was found in camels from Lower Egypt (19.92%) compared to the populations from Upper Egypt (7.78%). It is worth emphasizing that a greater number of studies have been conducted in Lower Egypt, and the prevalence of anti-*T. gondii* antibodies identified in our study aligns with the rates previously reported in this region (Table 1), although levels varied widely among studies. Using the same diagnostic method as the present study (MAT), similar seroprevalence value (17.46%; 29/166) was observed in a previous study (20) carried out in Lower Egypt (20). Another study assessed the potential efficacy of four antigen strains in the serodiagnosis of toxoplasmosis in 150 camels from Cairo using MAT and observed that the use of the *T. gondii* RH strain, and a local strain isolated from an equine provided seroprevalence rates of 18.00 and 20.66%, respectively, while a local camel and sheep strains provided seroprevalence rates of 27.33% (41/150) and 30.66% (46/150), respectively (21). On the other hand, several studies performed on a significant number of samples (more than 100 camel samples) using different serological methods, showed high seroprevalence levels (up to 64.51%) in camels in Lower Egypt (3, 21, 25, 26) (Table 1). In this respect, previous research using ELISA on camel blood samples in Lower Egypt revealed an overall seroprevalence rate of 46.9%, with specific rates of 39.16% (47/120) in Kafr El Sheikh, 45.00% (54/120) in Qalyubia, and 54.66% (82/150) in Marsa Matrouh (3). Another previous study conducted in Qalyubia governorate in Lower Egypt reported a high seroprevalence of 52.50% (63/120) using ELISA (8). In contrast, the same study (8) reported lower seroprevalence rates of 5.00% (6/120) for *T. gondii* in the same governorate using latex agglutination test (LAT) and indirect fluorescent antibody test (IFAT). The previously mentioned results showed important variations related to the method of serological test used. Among other techniques, MAT stands out as one of the most recommended for detection of *T. gondii* infection in both animals and humans (41). The test offers several advantages over other serological methods and is widely utilized due to its reliability and efficacy in detecting antibodies against the parasite. Additionally, MAT is recognized for its simplicity, cost-effectiveness, relatively high accuracy, and high sensitivity, making it a commonly used technique for serological detection of the parasite in different species, including camels (21).

It should be stressed that few studies have been conducted in Upper Egypt compared to Lower Egypt so far. Furthermore, none of the studies carried out in Upper Egypt used the MAT to detect antibodies against the parasite. The lowest *T. gondii* seroprevalence (0.81% 368 samples) in Upper Egypt was reported in the Red Sea governorate using ELISA (42). However, some studies, conducted in Aswan governorate, in Upper Egypt, on a smaller number of animals reported higher seroprevalence values (16, 26–28). In contrast to our findings, two previous studies (29, 30) on camels in Aswan governorate investigated the seroprevalence of *T. gondii* among a total of 37 and 92 animals, reporting seroprevalence rates of 16.30 and 32.43%, respectively, using LAT and ELISA (29, 30). Moreover, a serosurvey conducted on imported camels from Sudan, kept under two regions of Upper Egypt (Red Sea and Aswan), being distributed to other cities throughout the country, revealed significant differences in the seroprevalence between the two study sites (43). In this former study (43), a significantly higher seropositivity to *T. gondii* was recorded in the Red Sea camels (31.5%, 116/368) than in those sampled in Aswan (2.2%, 2/92). Two additional studies conducted among camels from Assiut governorate (Upper Egypt) documented higher prevalence rates of 15.00% (12/80) and 24.40% (30/119) for anti-*T. gondii* antibodies, using DT and LAT, respectively (16, 27). Another previous research (28) conducted on 56 camels in Assiut governorate (Upper Egypt) reported higher seroprevalence rates of 35.71 and 96.42% for *T. gondii* using LAT and ELISA, respectively (28).

Taken into consideration, various contributing factors could involve discrepancies in the spatial distribution of the parasite observed in the present work compared to previous studies at national or international levels. These factors include variations in environmental and climatic conditions, livestock management practices, and biosecurity measures. In certain governorates, camel management is based on traditional, extensive grazing with limited biosecurity measures. Meanwhile, others employ more commercial, urban-based systems with stricter biosecurity protocols, supported by better access to veterinary care and closer proximity to markets. Additionally, differences in the density of definitive hosts, sample sizes, serological methods, and the thresholds and sensitivities of tests and various stress factors that include grazing restrictions, limited movement, temperature changes, water availability, and nutrition further influence these variations (13, 40, 44–46). Meanwhile, discrepancies in seroprevalence values between the two study areas analyzed in the present study may be influenced by several factors including climatic variations encompassing temperature and humidity. In this regard, Lower Egypt typically experiences relatively lower temperatures and higher humidity compared to Upper Egypt (47). These ecological factors may favor the persistence of viable oocysts in the environment, thereby increasing the likelihood of exposure to the parasite (48, 49). Moreover, the higher density of definitive hosts, particularly cats, in the densely populated regions of Lower Egypt, especially Cairo, likely increases the risk of *T. gondii* exposure to other sympatric species including camels. In this respect, a serological study of stray cats in Cairo found an extremely high seroprevalence 95.55% of 180 cats analyzed by MAT (50). In contrast, there are no prior reports on the seroprevalence of *T. gondii* in cats in Upper Egypt, highlighting a significant gap in the research.

It is important to note that, although the age was not retained in the multivariate analysis, the bivariate analysis showed a significantly higher prevalence of *T. gondii* antibodies in camels over 4 years of age (Table 2), indicating a strong trend of increased seropositivity with advancing age. This finding aligns with various previous reports from Egypt (3, 26) and Ethiopia (51), reported higher seroprevalence among camels aged 8 years or older compared to younger ones. In contrary, other previous reports displayed no significant relation between the age of the camels and the seropositivity of *T. gondii* as (42). Considering that this trend has been observed across all mammals, this finding aligns with those previously observed in this species that reflects a cumulative likelihood for exposure to *T. gondii* and lifelong persistence of antibodies (3, 26, 51). It should be noted that adult animals, having lived longer, are exposed to a wider array of infection sources compared to younger animals, accounting for the higher seroprevalence rates observed in older animals (52, 53). Collectively, the above-mentioned findings suggest that horizontal transmission might be considered the main route of *T. gondii* infection in camels.

## 5 Conclusion

The current study offers updated insights into the exposure of dromedary camels to *T. gondii* across Upper and Lower Egypt. Our findings revealed a widespread circulation and heterogenous spatial distribution of this protozoan in the camel populations in this country which might have important health implication for this species. While the presence of antibodies against *T. gondii* does not confirm whether the host harbors viable parasites, the findings of this study highlight the potential risk of zoonotic transmission from camels, particularly through the consumption of raw or undercooked camel milk and meat. Further large-scale serosurvey and molecular investigations are warranted to assess the role of this species in the epidemiology of *T. gondii* in Egypt. Control measures should be implemented to minimize the risk of camel exposure to this zoonotic parasite. Along with raising public health awareness, these measures should encompass the application of proper farm management practices, preventing contamination of feed and water by *T. gondii*-infected cats, the control of stray cat populations and their access to animals premisses, and conducting regular testing of animals at large scale level.

## Data Availability

The original contributions presented in the study are included in the article/supplementary material, further inquiries can be directed to the corresponding authors.
